# Myocardial infarct size is reduced by nitrite and nitrate administration: a systematic review and meta-analysis of animal studies

**DOI:** 10.17179/excli2023-6740

**Published:** 2024-01-03

**Authors:** Younes Yassaghi, Sajad Jeddi, Khosrow Kashfi, Asghar Ghasemi

**Affiliations:** 1 Endocrine Physiology Research Center, Research Institute for Endocrine Sciences, Shahid Beheshti University of Medical Sciences, Tehran, Iran; 2 Department of Molecular, Cellular, and Biomedical Sciences, Sophie Davis School of Biomedical Education, City University of New York School of Medicine, NY, USA

**Keywords:** nitrite, nitrate, myocardial infarction, ischemia-reperfusion injury, infarct size, cardioprotection

## Abstract

Ischemic heart disease (IHD) is the leading cause of mortality worldwide and can be complicated by myocardial infarction (MI), leading to cardiac failure. Inorganic nitrite and nitrate, which release nitric oxide (NO), can protect the heart against myocardial injury. This animal systematic review and meta-analysis aims to assess whether the administration of nitrite/nitrate decreases myocardial infarct size. We systematically searched PubMed, Scopus, and Web of Science databases until October 2023; 15 eligible animal studies (35 study arms for in-vivo and 10 for in-vitro studies) published between 1989 and 2023 were included. *In-vivo* studies were conducted on rats, mice, cats, and dogs, and *in-vitro* studies on rats and mice with an overall exposure of 0.03 to 12713 mg/kg to nitrate/nitrite administrated before, after, or during ischemia mainly by intravenous single bolus or by oral over 270 days. All *in-vitro* studies used nitrite/nitrate before ischemia, with the concentration ranging between 0.34 to 201 μM. MI was induced by occlusion of the left anterior diagonal or left circumflex arteries in *in-vitro* studies and by isoproterenol in *in-vivo* studies. Infarct size was measured by direct staining of the sliced heart sections. In *in-vivo* studies, nitrite (overall effect size (ES)=-17.0 %, 95 % confidence interval (CI)=-21.3, -12.8, P<0.001) and nitrate (overall ES= -9.6 %, 95 % CI=-15.7, -3.4, P=0.002) reduced myocardial infarct size. In *in-vitro* studies, nitrite (overall ES=-15.8 %, 95 % CI=-25.5, -6.2, P=0.001) reduced the infarct size. Sensitivity analysis showed that the overall effect of nitrite on myocardial infarct size was unaffected by doses or health conditions in *in-vivo* and *in-vitro* studies. In conclusion, our meta-analysis showed that nitrite/nitrate administration can effectively reduce myocardial infarct size. However, these results should be approached with caution because of the limitations of animal studies and the existing high heterogeneity.

## Introduction

Ischemic heart disease (IHD) is the leading cause of burden and mortality worldwide (Safiri et al., 2022[37]). The global prevalence of IHD was 244.1 million in 2020 (Tsao et al., 2022[48]), which is estimated to be increased by 11.5 % by 2030 (Khan et al., 2020[27]). The global prevalence of myocardial infarction (MI), an event primarily due to IHD, between 1976 and 2022 was 3.8 % and 9.5 % in subjects younger and older than 60 years, respectively (Salari et al., 2023[38]). The standard management of MI is pharmacotherapy followed by emergent percutaneous coronary intervention (PCI) or coronary artery bypass graft surgery for revascularization (Lawton et al., 2022[29]). However, this management can lead to secondary myocardial damage called reperfusion injury, further complicating the situation (Heusch, 2020[15]). The two-year mortality rate of MI was 2.7 % in Eastern and 8.1 % in Western countries despite treatment (Rosselló et al., 2017[36]). Therefore, additional preventive and therapeutic approaches for MI management and myocardial ischemia-reperfusion injury (MIRI) are warranted. 

Nitric oxide (NO) is a gasotransmitter with cardioprotective effects (Johnson et al., 1991[22]). In the heart tissue, NO is mostly (~80 %) produced by the NO synthase (NOS) enzymes and, to a lesser extent (~20 %), by the nitrate-nitrite-NO pathway (Ghasemi and Jeddi, 2022[9]). During myocardial ischemia, the expression (Jeddi et al., 2016[20]) and activity (Giraldez et al., 1997[12]) of the endothelial NOS (eNOS), which is the major contributor to the heart NO production in physiological conditions (Ghasemi and Jeddi, 2022[9]), are diminished, and NO is predominantly produced by the nitrate-nitrite-NO pathway (Samouilov et al., 1998[40]). Administration of inorganic nitrite and nitrate, compounds that release NO in the human body (Lundberg et al., 2015[31]), affect heart NO availability and the risk of myocardial injury. The cardioprotective effects of nitrite and nitrate have been reported in mice (Bryan et al., 2007[4]; Salloum et al., 2015[39]) and rats (Baker et al., 2007[3]; Jeddi et al., 2016[20]). Acute intraperitoneal (IP) injection of sodium nitrite in rats (1 mg/kg) (Bryan et al., 2005[6]) and mice (10 mg/kg) (Almeida et al., 2015[1]) increased heart tissue nitrite concentration. A 7-day low-nitrite diet in mice decreased heart nitrite concentration by ~80 % and aggravated the MIRI; a subsequent 7-day nitrite supplementation (50 mg/L in drinking water) restored the nitrite concentration to control values and reversed the aggravated MIRI (Bryan et al., 2007[4]). In addition, vegetable-rich diets, contributing to 60-80 % of overall nitrate intake (Weitzberg and Lundberg, 2013[50]), reduce the risk of IHD (Joshipura et al., 2001[25]) and its mortality (Jabri et al., 2021[19]). The mortality rate due to MI after treatment is lower in Eastern countries (Rosselló et al., 2017[36]), where vegetable intake is higher than in Western countries (349 g/day in East Asia compared to 56 g/day in Central America among the data of 188 countries) (Kalmpourtzidou et al., 2020[26]). 

Myocardial infarct size (the percentage of the area with hyper-enhanced cardiac magnetic resonance imaging to total left ventricle (LV) area (Wu et al., 2008[51])) is the primary endpoint used in clinical studies to assess the effect of ischemia on the heart (Thiele et al., 2008[45]). The reason is that myocardial infarct size has a more robust capability over hemodynamic measurements, such as ejection fraction, for predicting adverse outcomes of MI (Wu et al., 2008[51]). A meta-analysis of clinical studies showed that for each 5 % increase in myocardial infarct size after vascular intervention, mortality is 19 % more likely to occur (hazard ratio (HR)=1.19 %, confidence interval (CI)=1.18, -1.2) (Stone et al., 2016[44]). Some studies indicated that administration of nitrite and nitrate decreases infarct size in mice (Bryan et al., 2007[4]); however, other studies have reported that nitrate in rats (Baker et al., 2007[3]) and nitrite in mice (Hendgen-Cotta et al., 2008[14]) do not decrease myocardial infarct size. In addition, a study in cats has reported that nitrite slightly increases (~2.8 %) infarct size (Johnson et al., 1991[22]). This animal systematic review and meta-analysis aims to answer this question: Does the administration of nitrate/nitrite decrease myocardial infarct size? 

## Methods

We followed the Preferred Reporting Items for Systematic Reviews and Meta-Analyses (PRISMA) 2020 updated guideline (Page et al., 2021[33]) to design and perform this study and analyze and report the results.

### Search strategy and study selection 

Databases, including PubMed, Scopus, and Web of Science, were searched until October 2023 by the combination of the following search terms: (nitrate, nitrite, nitrogen dioxide, nitric oxide, sodium nitrite, sodium nitrate, or beetroot) and (reperfusion injury, ischemia/reperfusion, ischemia-reperfusion, ischemia reperfusion, myocardial ischemia, cardiac ischemia, heart ischemia, myocardial infarction, or cardioprotection) (Supplementary Table 1). 

**Figure 1:**
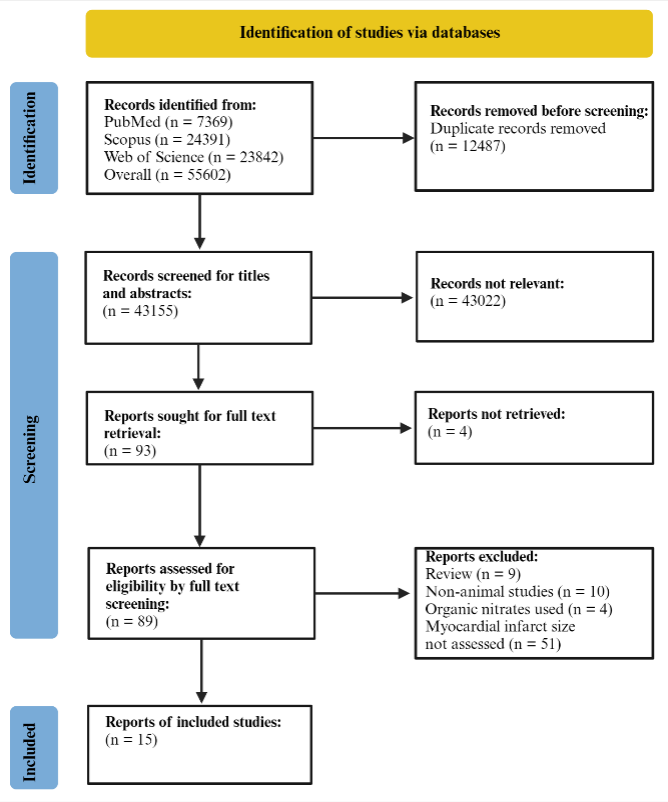
PRISMA flow diagram of the included studies. Created with BioRender.com

The records were extracted from each database to Endnote version 18 software, and the duplicate records were removed. The remaining records were screened for titles and abstracts in the first screening step, and the non-relevant records were excluded. Full texts of the relevant studies were then sought and screened cautiously and thoroughly for their eligibility. All animal studies published in English that assessed the effect of inorganic nitrite or nitrate administration on MI or MIRI were included. Non-animal studies (clinical studies, observational studies, non-interventional *in-vitro* studies), studies on organs excluding the heart, studies that used organic nitrates, review articles (including systematic reviews and meta-analyses), conference abstracts, comments, letters to the editor, symposiums, studies without accessible full texts, and studies that did not report myocardial infarct size were excluded. Eventually, eligible studies (15 studies with 35 *in-vivo* study arms and 10 *in-vitro* study arms) were considered for final inclusion (Figure 1(Fig. 1)). The references of the final included studies were also screened to reduce the risk of missing relevant studies. 

### Quality assessment and risk of bias

To evaluate the existing risk of bias and the quality of the included studies, we used the Systematic Review Center for Laboratory Animal Experimentation Risk of Bias Tool (SYRCLE's RoB tool) provided by Hooijmans et al. (2014[17]). Briefly, studies were assessed by two authors independently, and a third author further discussed the disagreements. Each author answered ten questions about different types of bias in each study by scoring 1 (no existing bias) or 0 (existing bias) on every question, resulting in a 0 to 10 score for each study's bias risk; a higher score indicates higher study quality and lower study bias while a lower score indicates the opposite. The SYRCLE's RoB score is reported in the table of characteristics for each study individually (Table 1(Tab. 1); References in Table 1: Baker et al., 2007[3]; Bryan et al., 2007[4], 2008[5]; Gonzalez et al., 2008[13]; Hendgen-Cotta et al., 2008[14]; Johnson et al., 1990[23][21], 1991[22]; Raish et al., 2019[35]; Salloum et al., 2015[39]; Shiva et al., 2007[41]; Totzeck et al., 2017[46]; Tripathi et al., 1997[47]; Yassaghi et al., 2023[52]; Table 2(Tab. 2); References in Table 2: Baker et al., 2007[3]; Hendgen-Cotta et al., 2008[14]; Webb et al., 2004[49]), and all studies' mean SYRCLE's RoB score was calculated.

### Data extraction 

Information extracted from each study included the first author's name, year of publication, animal species, animal sex, health condition of animals, number of animals in the treatment and control groups, model of the experiment (MI or MIRI), type of ischemia (global or regional), type of intervention (nitrite or nitrate), calculated overall exposure dose, as well as route, time, and duration of administration. The mean and standard deviation (SD) of each myocardial infarct size were also extracted. If a study had measured the myocardial infarct size following several doses and/or treatment durations, each measurement was considered a separate experimental study. These data were extracted from the main article texts or table of results; if a study had represented the results only as a graph, the data was extracted using Adobe Photoshop software using a previously described method by Gheibi et al. (2019[11]). 

### Statistical analysis

To conduct statistical analysis and illustrations of this meta-analysis study, we used STATA software, version 17. The random effect model of meta-analysis was used because it is preferred over the fixed effect model in cases of considerable heterogeneity. For the assessment of the overall effect of nitrite and nitrate administration on myocardial infarct size, weighted mean difference (WMD) was used instead of standard mean difference as the ES since all of the included studies had reported the myocardial infarct size as the percentage of the infarcted area of the LV to total area at risk (AAR). The overall ES was analyzed for *in-vivo* and *in-vitro *studies separately for each treatment (nitrite or nitrate) and reported with a 95 % confidence interval (CI); the results were considered statistically significant for the probability levels (P-value) <0.05. We also conducted a sensitivity analysis by excluding studies that administered very high doses of nitrite (including 3178.3 (Johnson et al., 1990[23][21]), 6356.6 (Johnson et al., 1990[23]), and 12713.3 mg/kg (Johnson et al., 1990[23], 1991[22])) and that used non-healthy conditioned (such as eNOS-/- (Bryan et al., 2008[5]) and myoglobin-/- (Hendgen-Cotta et al., 2008[14])) animals. 

To assess whether the outcomes of the studies are consistent, heterogeneity was calculated by I^2^ (I-squared) as the percentage of heterogeneity that indicates the extent of the studies that the CI of their outcome does not overlap with the overall ES, and Cochran's Q statistics (also called the test of homogeneity) representing the probability that the outcomes of the studies are homogenous (Higgins et al., 2003[16]). The results of I^2 ^are reported and interpreted according to the following categories: 0-25 %=no heterogeneity, 25-50 %=low heterogeneity, 50-75 %=moderate heterogeneity, and >75 %=high heterogeneity. For Cochran's Q test, the P-value of the homogeneity test was reported and interpreted as heterogeneous outcomes if P<0.1. 

The existing heterogeneity was investigated by a random effect meta-regression for the overall exposure dose and a subgroup analysis for the following covariates: animal species and type of ischemia for *in-vitro* and animal species, route of administration, time of administration, and duration of administration for *in-vivo* studies. For meta-regression, the R^2^ was reported, indicating the amount of heterogeneity explained by the moderator; if the overall exposure dose was not available for a study, the study was excluded from the meta-regression analysis. In subgroup analysis, the possible sources of heterogeneity were predetermined before facing a high heterogeneity result to avoid false-positive results. To define the sources of heterogeneity, aside from a significant test of group differences (P<0.1), other factors were considered, including comparable distribution of studies between the subgroups, the plausibility of the interaction, the importance of the interaction, and the possibility of confounding covariates (Siervo et al., 2011[43]). Furthermore, the result of the subgroup analysis is reported as quantitative (all overall effect sizes are on the same side of the null-effect line) or qualitative (in the opposite direction) (Siervo et al., 2011[43]). 

Due to the variations in the overall exposure doses between the studies, dose-response analysis was not performed in our analyses. For a more precise evaluation of treatment effects, the results of the studies were analyzed for nitrite and nitrate administration separately within *in-vivo* and *in-vitro* studies.

## Results

### Characteristics of the included studies 

The number of records obtained from our systematic search through databases was 43115 after omitting the duplicates. After screening titles and abstracts, 43022 records were excluded, leaving 93 records for full-text screening. After retrieving and screening the full texts, 15 studies were considered eligible for inclusion and read thoroughly for data extraction. 

As is seen in Table 1(Tab. 1), the *in-vivo* studies were published from 1989 to 2023, including 14 studies and 35 study arms with an overall 225 treatment and 229 control animals. Studies used different animal species (rats, mice, dogs, and cats), with rats being the most used animal in the study arms. Except for five study arms with no reports of animal sex (Bryan et al., 2007[4], 2008[5]; Gonzalez et al., 2008[13]) and one study that used female rats (Yassaghi et al., 2023[52]), all other arms had used male animals. All animals used in the studies were healthy, except two study arms that used myoglobin^-/- ^(Hendgen-Cotta et al., 2008[14]) and eNOS^-/-^ (Bryan et al., 2008[5]) mice. Studies used MI or IR as the ischemia model, of which IR was used the most in the study arms. The treatment (nitrite or nitrate) was administered by different routes, including IP, intraventricular (heart ventricle; IVT), intravenous (IV), and oral administrations, of which the IV route was used in most study arms, and with nitrite being used more than nitrate. Since the onset and duration of the administration were wide and variant, we categorized the onset of the administration as before, during, and after ischemia, and the duration of administration as a bolus (one injection or oral gavage), less than 24 hours (infused from minutes to hours but not more than one day), and more than 24 hours (more than one day), of which the treatment was mostly administered before ischemia and by a single bolus dose. In addition, the range of the overall exposure dose was 0.03 to 12713.3 mg/kg.

*In-vitro* studies were published from 2004 to 2008, including three studies and ten study arms with 60 treatment and 64 control animals (Table 2(Tab. 2)). Studies used rats and mice as experimental models, of which rats were used more than mice. All used animals were males and with healthy conditions except one study arm that used male myoglobin^-/-^ mice (Hendgen-Cotta et al., 2008[14]). The ischemia model used in these studies was IR with regional and global types, of which regional type was the most used ischemia type. Except for one study arm that used nitrate (Baker et al., 2007[3]), all others used nitrite for treatment. All treatments were administered before the induction of ischemia with an overall exposure dose of 0.34 to 201 µM.

### Publication bias and quality assessment 

Funnel plots for nitrite *in-vivo* and *in-vitro* studies and nitrate *in-vivo* studies showed a symmetric distribution of the study arms considering overall ES, indicating a low publication bias. Also, Egger's regression test showed no evidence of publication bias in our study.

The mean SYRCLE's RoB score of the study arms was 4.3 for *in-vivo* and 4.2 for *in-vitro* studies, indicating a moderate quality and risk of bias overall. Selection bias (due to allocation concealment), performance bias (due to random housing and blinding), and detection bias (due to random outcome assessment) were present in all studies. In addition, except for two studies (Salloum et al., 2015[39]), all other studies had selection bias due to allocation sequence generation. Also, only five studies (Johnson et al., 1990[23]; Bryan et al., 2008[5]; Gonzalez et al., 2008[13]; Hendgen-Cotta et al., 2008[14]; Totzeck et al., 2017[46]) had reported the blindness of the outcome assessor, and a detection bias (blindness of the outcome assessor) was present in other studies. 

### In-vivo studies

**Figure 2:**
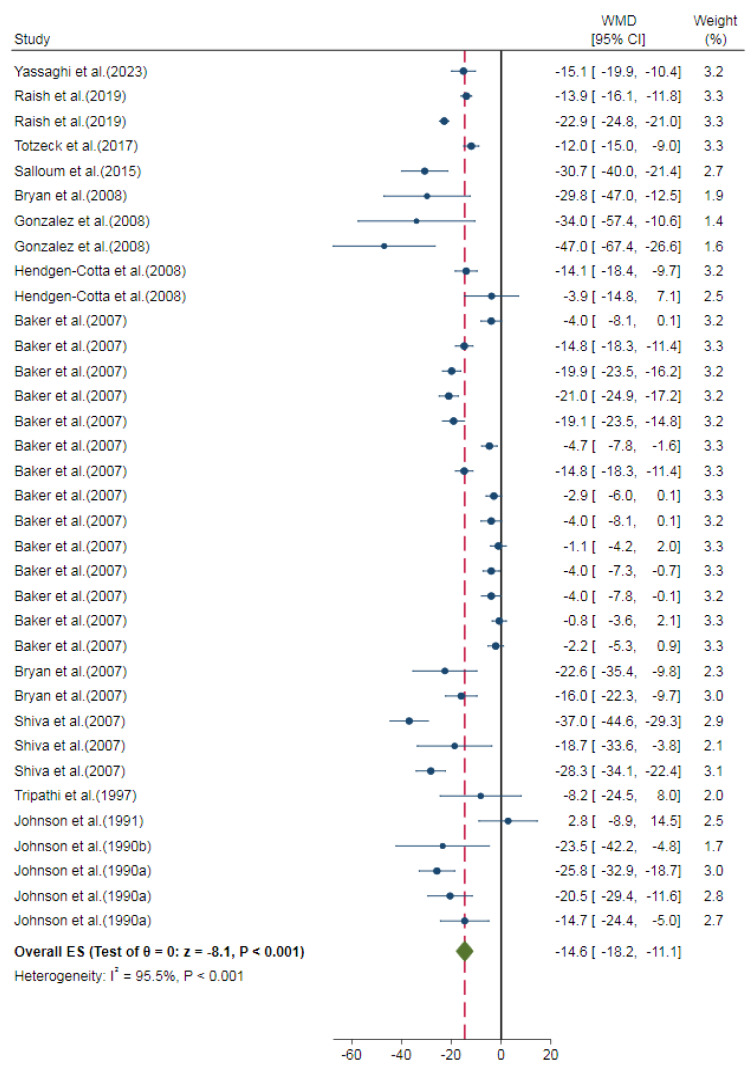
Overall effect size (ES) of the nitrite/nitrate administration on myocardial infarct size in *in-vivo* studies

**Figure 3:**
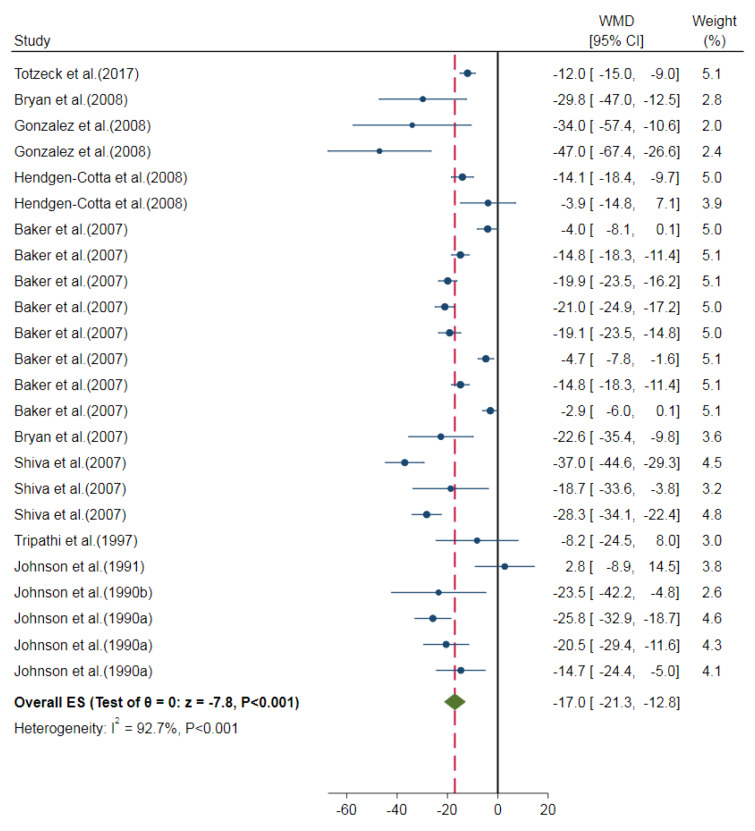
Overall effect size (ES) of the nitrite administration on myocardial infarct size in *in-vivo* studies

Overall, nitrite/nitrate administration decreased the myocardial infarct size compared to control groups in *in-vivo* studies (overall ES=-14.6 %, 95 % CI=-18.2, -11.1, P<0.001). The heterogeneity of the results was high (I^2^= 95.5 %) (Figure 2(Fig. 2); References in Figure 2: Baker et al., 2007[3]; Bryan et al., 2007[4], 2008[5]; Gonzalez et al., 2008[13]; Hendgen-Cotta et al., 2008[14]; Johnson et al., 1990[23][21], 1991[22]; Raish et al., 2019[35]; Salloum et al., 2015[39]; Shiva et al., 2007[41]; Totzeck et al., 2017[46]; Tripathi et al., 1997[47]; Yassaghi et al., 2023[52]). In addition, a smaller myocardial infarct size was observed in nitrite-treated animals compared to control groups (overall ES=-17.0 %, 95 % CI=-21.3, -12.8, P<0.001) with a high heterogeneity among study outcomes (I^2^= 92.7 %) (Figure 3(Fig. 3); References in Figure 3: Baker et al., 2007[3]; Bryan et al., 2007[4], 2008[5]; Gonzalez et al., 2008[13]; Hendgen-Cotta et al., 2008[14]; Johnson et al., 1990[23][21], 1991[22]; Raish et al., 2019[35]; Salloum et al., 2015[39]; Shiva et al., 2007[41]; Totzeck et al., 2017[46]; Tripathi et al., 1997[47]; Yassaghi et al., 2023[52]). To investigate this high heterogeneity, subgroup analysis was performed for predetermined covariates. The overall ES was not different between the subgroups for animal species and the duration of administration. However, different routes of administration (P<0.001) and the onset of administration (P<0.001) had significantly different overall ES (Table 3(Tab. 3)). In addition, meta-regression analysis showed no role for the overall exposure in heterogeneity (R^2^<0.001; one study did not report the dose of administration (Tripathi et al., 1997[47]) and was excluded from meta-regression). The results of the sensitivity analysis showed no considerable change after excluding studies with very high doses (overall ES=-17.3 %, 95 % CI=-22.1, -12.4, P<0.001) (Johnson et al., 1990[23][21], 1991[22]) or non-healthy conditions (overall ES=-17.2 %, 95 % CI=-21.6, -12.8, P<0.001) (Bryan et al., 2008[5]; Hendgen-Cotta et al., 2008[14]).

**Figure 4:**
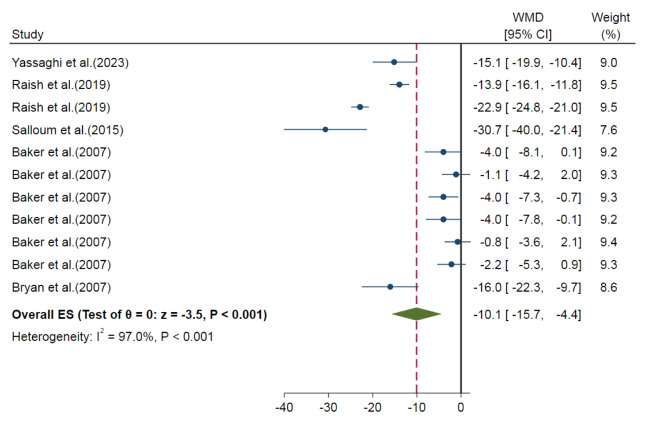
Overall effect size (ES) of the nitrate administration on myocardial infarct size in *in-vivo* studies

Our meta-analysis showed that nitrate-administered animals also had smaller myocardial infarct size compared to control groups (overall ES=-10.1 %, 95 % CI=-15.7, -4.4, P<0.001) with a high heterogeneity among studies (I^2^= 97.0 %) (Figure 4(Fig. 4); References in Figure 4: Baker et al., 2007[3]; Bryan et al., 2007[4]; Raish et al., 2019[35]; Salloum et al., 2015[39]; Yassaghi et al., 2023[52]). The test of group differences was statistically significant between the subgroups for animal species (P=0.049), the duration of administration (P<0.001), and the route of administration (P<0.001) (Table 3(Tab. 3)). However, the route and duration of administration can be considered as one subgroup since all oral nitrate administrations were delivered more than 24 hours, and all IV administrations were delivered as a single bolus dose. Meta-regression analysis showed no role for the overall exposure in the heterogeneity (R^2^<0.001).

### In-vitro studies

**Figure 5:**
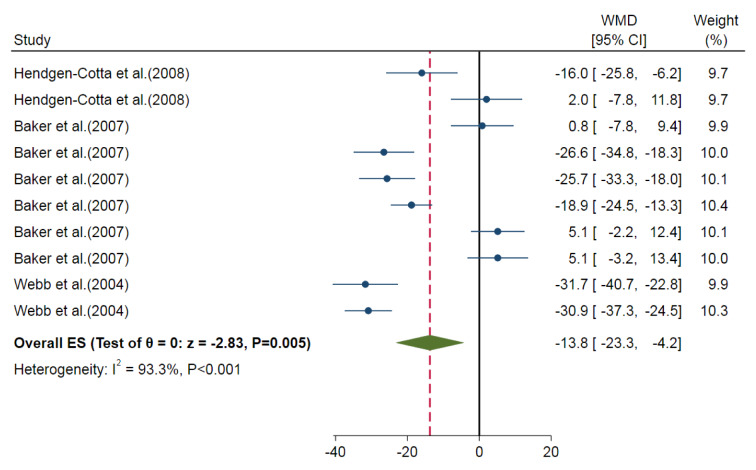
Overall effect size (ES) of the nitrite/nitrate administration on myocardial infarct size in *in-vitro* studies

**Figure 6:**
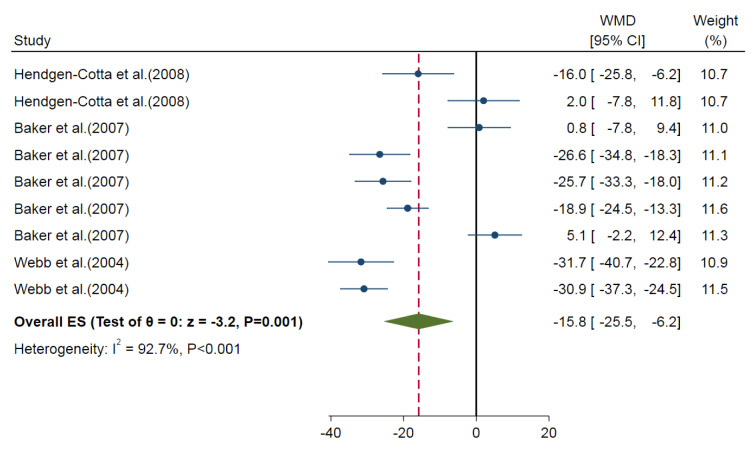
Overall effect size (ES) of the nitrite administration on myocardial infarct size in *in-vitro* studies

Our meta-analysis showed a smaller myocardial infarct size for nitrite/nitrate administration compared to control groups (overall ES=-13.8 %, 95 % CI=-23.3, -4.2, P=0.005) in *in-vitro* studies. The calculated heterogeneity was high (I^2^= 93.3 %, P<0.001) (Figure 5(Fig. 5); References in Figure 5: Baker et al., 2007[3]; Hendgen-Cotta et al., 2008[14]; Webb et al., 2004[49]). In addition, nitrite-administered animals had smaller myocardial infarct sizes (overall ES=-15.8 %, 95 % CI=-25.5, -6.2, P=0.001) with high heterogeneity (I^2^=92.7 %) (Figure 6(Fig. 6); References in Figure 6: Baker et al., 2007[3]; Hendgen-Cotta et al., 2008[14]; Webb et al., 2004[49]). We could not analyze the results of the nitrate administered *in-vitro* studies since there was only one *in-vitro* study arm (Baker et al., 2007[3]). Subgroup analysis was performed for animal species (P=0.290) and the type of ischemia (P=0.001) (Table 4(Tab. 4)). Also, meta-regression analysis showed no involvement of the overall exposure in the heterogeneity (R^2^<0.001). Omitting one study arm with a non-healthy condition (myoglobin^-/-^) (Hendgen-Cotta et al., 2008[14]) increased ES (overall ES=-18.0 %, 95 % CI=-27.7, -8.3, P<0.001).

## Discussion

In this study, we systematically reviewed and meta-analyzed the data from 45 animal study arms on the effect of nitrite/nitrate administration on myocardial infarct size. Overall, our analyses showed that nitrite/nitrate administration decreased myocardial infarct size by approximately 10 % to 17 % (roughly 15 %) in *in-vivo* and *in-vitro* studies. Sensitivity analysis showed that the overall effect of nitrite on myocardial infarct size was unaffected by doses or health conditions in *in-vivo* and *in-vitro* studies. 

The mechanism underlying the protective effects of nitrite/nitrate against MI or MIRI has been discussed in several studies. Restoration and increased concentrations of circulating and heart tissue nitrite, nitrate, and NOx (nitrite+nitrate) have been related to the protective effects of nitrite and nitrate on infarct size (Bryan et al., 2007[4], 2008[5]). In addition, the deoxymyoglobin (Hendgen-Cotta et al., 2008[14]) and xanthine oxidoreductase (Webb et al., 2004[49]; Baker et al., 2007[3]) mediated reduction of nitrite to NO leads to diminished production of reactive oxygen species (ROS) (Hendgen-Cotta et al., 2008[14]) by S-nitrosylation and inhibition of mitochondrial complex I (Shiva et al., 2007[41]) and various other proteins, including calpains (activation of which results in cellular death) (Totzeck et al., 2017[46]), which decreases myocardial infarct size (Webb et al., 2004[49]; Baker et al., 2007[3]; Shiva et al., 2007[41]; Hendgen-Cotta et al., 2008[14]; Totzeck et al., 2017[46]). Moreover, nitrite decreases myeloperoxidase (MPO) activity, indicating the inhibition of neutrophil accumulation and inflammation (Johnson et al., 1990[23]). Beetroot juice (BRJ, containing high amounts of nitrate (Hord et al., 2009[18])) decreases infarct size by cystathionine-γ-lyase (CSE)-dependent increases in hydrogen sulfide (H_2_S) levels in heart tissue (Salloum et al., 2015[39]), blunting the decrease in NO levels (Raish et al., 2019[35]), diminishing ROS production and levels of malondialdehyde (inducing lipid peroxidation and oxidative stress), and inflammatory products (NF-κB, TNF-α, and IL-6) (Raish et al., 2019[35]). Also, nitrate has been shown to decrease myocardial infarct size by blunting the decrease in eNOS and increase in iNOS expressions (Yassaghi et al., 2023[52]).

Results of subgroup analysis showed that different durations of administration for nitrate in *in-vivo* studies changed the overall ES significantly, of which oral administration for more than one day resulted in a significantly smaller myocardial infarct size compared to an IV bolus dose. In addition, except for two studies (Bryan et al., 2007[4], 2008[5]), nitrite was administered acutely (bolus and <24 hours). This indicates that nitrate is more effective when administered for longer durations and can be considered a preventive preconditioning agent, and nitrite can be used for both the prevention and the treatment of myocardial injury. These results further support the findings of Bryan et al. (2005[6]) that showed acutely administered nitrite is metabolized to nitroso and nitrosyl products, increasing nitrite concentration in different tissues, including the heart. In contrast, this was not the case for nitrate. Furthermore, subgroup analysis showed that nitrate significantly decreases myocardial infarct size in mice more than in rats. This may be due to physiological differences between these species, as total NO production in mice is reported to be about 15 times more than in rats (7.68±1.47 versus 0.55±0.05 µmol/kg per hr) (Siervo et al., 2011[43]).

Additionally, subgroup analysis for the onset of administration showed that nitrite administration before and during the ischemia period resulted in smaller myocardial infarct size than administration after ischemia. This supports the results of the clinical study by Jones et al. (2015[24]) that administered intracoronary nitrite before reperfusion in patients undergoing PCI is warranted. They showed that nitrite reduces myocardial infarct size in patients with complete artery occlusion but not in patients with partial coronary flow. Moreover, our results showed that IV administration of nitrite reduces myocardial infarct size in *in-vivo* studies (Table 3(Tab. 3)), which was in contrast with the results of a clinical trial by Siddiqi et al. (2014[42]) that showed no effect of IV nitrite administration before reperfusion on infarct size. This may be due to the different pharmacokinetics of nitrite in animals and humans since similar doses of a study by Gonzalez et al. (2008[13]) in dogs did not increase serum nitrite levels in humans as the same (Siddiqi et al., 2014[42]). For *in-vitro* studies, subgroup analysis showed that nitrite significantly reduces myocardial infarct size in global ischemia more than regional ischemia. This may be due to the methodological differences between global and regional ischemia models as it has been shown that *in-vitro* 30 min global ischemia followed by 2 hours reperfusion results in larger infarct size compared to regional ischemia; however, regional ischemia is more relevant than global ischemia to the clinical situation (Kim et al., 2012[28]).

Our study has some limitations; first, the analyses in this study showed a considerably high heterogeneity between study outcomes. Although subgroup analysis highlighted the onset and route of administration for nitrite in *in-vivo* studies, animal species and route or duration of administration for nitrate in *in-vivo* studies, and type of ischemia for nitrite in *in-vitro* studies as sources of heterogeneity, these cannot reliably be considered as the sources of heterogeneity since there was no comparable distribution of studies between the subgroups. Second, the studies included in our meta-analysis were heterogeneous in designs, study subjects, dose, timing and route of administrations, and these variations of designs between studies may be the cause of the present heterogeneity in our study. This also makes it difficult for meta-analysis of animal studies to translate into clinical purposes, and it is proposed that these meta-analysis studies may be helpful for making hypotheses and helping design clinical studies (Bahadoran et al., 2020[2]). As is seen in our results, the included studies had a methodological bias in several aspects, including allocation concealment, random housing, investigator/outcome assessor blinding, random outcome assessment, and allocation sequence generation. Therefore, it has been suggested that animal studies should comply with existing guidelines for the design and report of animal studies (Ghasemi and Jeddi, 2023[10]), such as Harmonized Animal Research Reporting Principles (HARRP) (Osborne et al., 2018[32]) and Animal Research: Reporting of *In-Vivo* Experiments (ARRIVE) (Percie du Sert et al., 2020[34]) to avoid the variations in study designs. Finally, there was a clear male bias in the included studies despite the recommendation of the National Institutes of Health (NIH) to use both male and female animals in the experiments (Clayton and Collins, 2014[7]).

As a strength, we considered myocardial infarct size as the outcome of the cardioprotection of nitrite/nitrate since it is more useful for predicting the outcomes of MI than other cardiac measurements (Wu et al., 2008[51]), and every 5 % increase in the myocardial infarct size results in a 19 % increase in mortality (Stone et al., 2016[44]). Moreover, the weight of the included studies was roughly even in different analyses, indicating that the overall ES obtained in our study was not related to specific studies and is not a false-positive effect.

## Conclusion

Overall, the results of this meta-analysis in animals showed that nitrite/nitrate administration can reduce myocardial infarct size by approximately 15 %. It might be more effective if nitrate is used as a preconditioning agent to prevent myocardial injury. Indeed, nitrate has been suggested to be used as a dietary supplement (like table salt) for cardioprotection (Lundberg et al., 2018[30]). On the other hand, nitrite is more effective as a treatment when facing myocardial ischemia. Although the results of our study are promising, they should be approached with caution since meta-analyses of animal studies often fail to be translated into human data due to the limitations of animal studies (Bahadoran et al., 2020[2]).

## Declaration

### Acknowledgments and funding information

This study was supported by a grant (Grant No. 43007749-4) from Shahid Beheshti University of Medical Sciences.

### Declaration of competing interest 

The authors declare that they have no competing interests.

### Authorships

Younes Yassaghi and Sajad Jeddi contributed to screening and quality assessment of studies and data extraction. Younes Yassaghi contributed to the literature review and wrote the article. Sajad Jeddi, Khosrow Kashfi, and Asghar Ghasemi provided critical revision and final approval of the finalized manuscript. All authors have read and approved the final manuscript.

## Supplementary Material

Supplementary information

## Figures and Tables

**Table 1:**
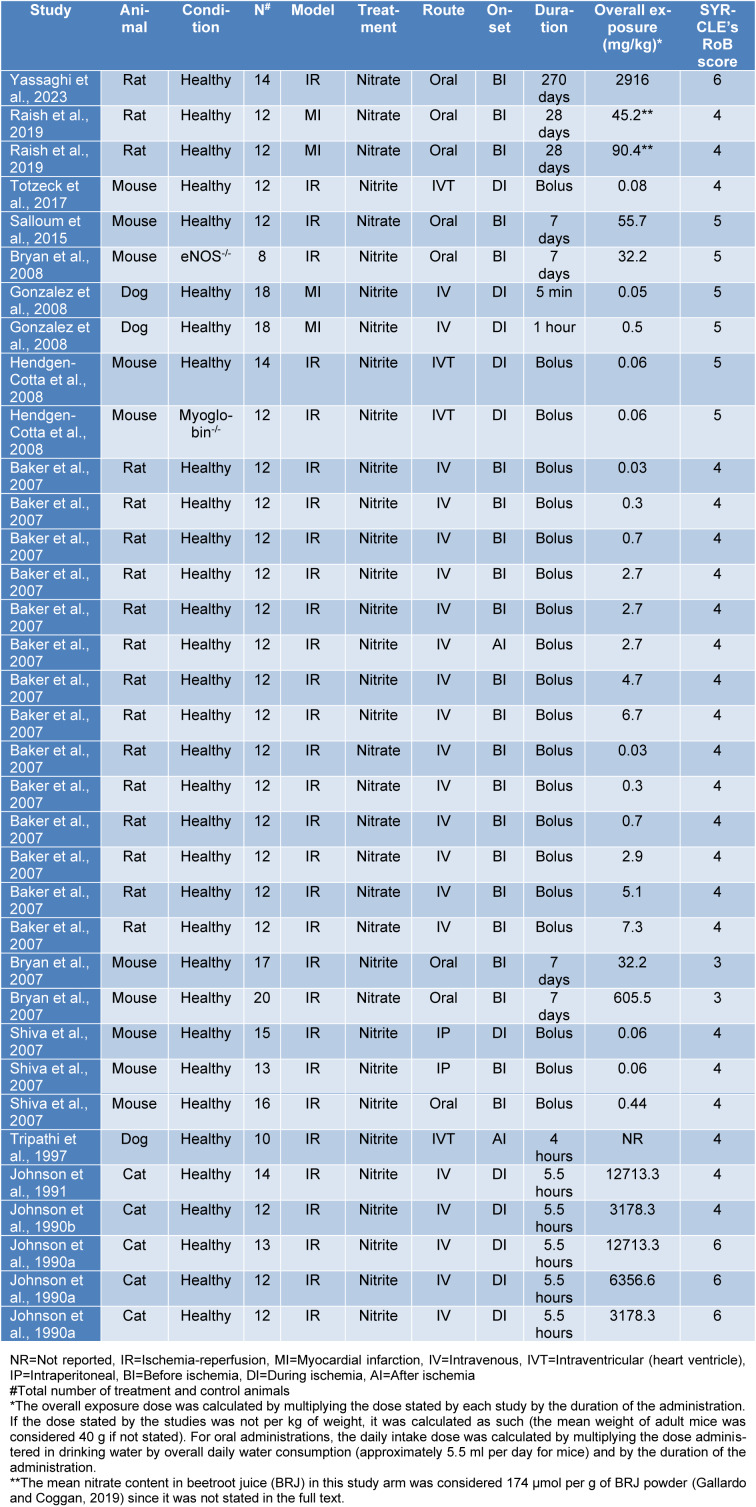
Characteristics of *in-vivo* studies

**Table 2:**
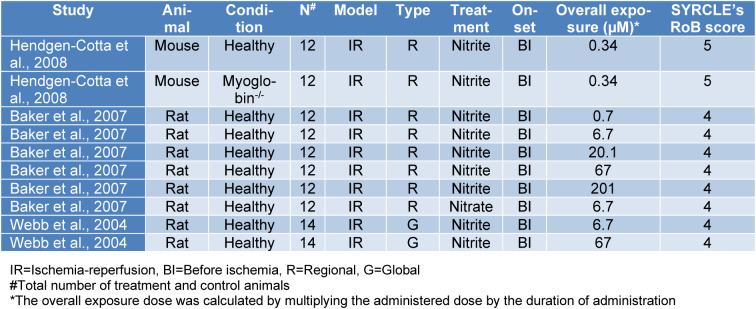
Characteristics of *in-vitro* studies

**Table 3:**
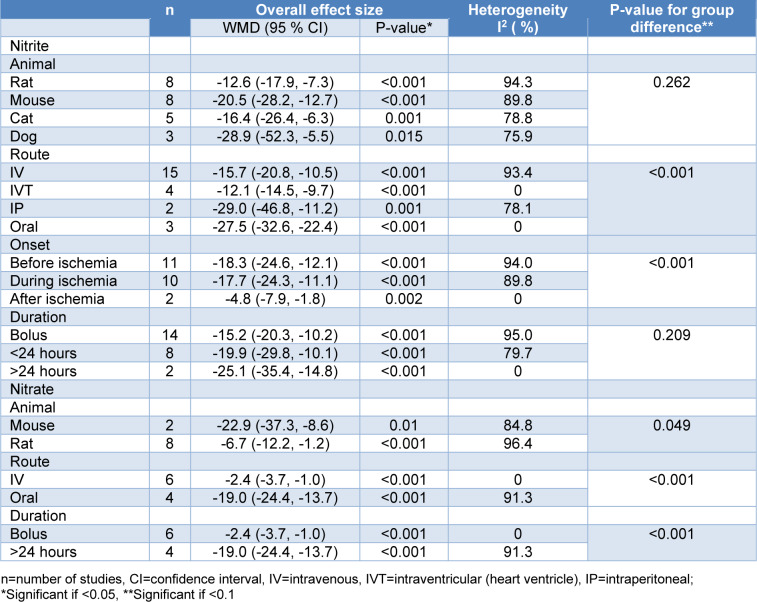
Subgroup analysis of the weighted mean difference (WMD) of myocardial infarct size between treatment and control groups in *in-vivo* studies

**Table 4:**
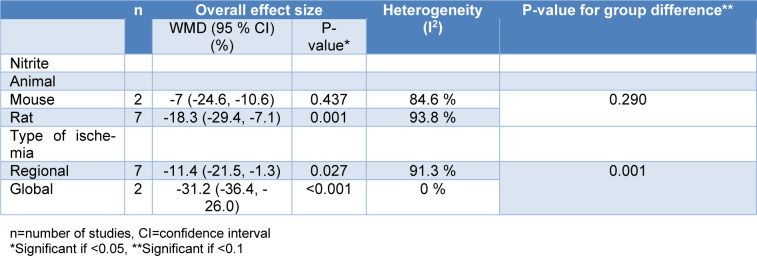
Subgroup analysis of the weighted mean difference (WMD) of myocardial infarct size between treatment and control groups in *in-vitro* studies
