# Supplementary material for: Myocardial infarct size is reduced by nitrite and nitrate administration: a systematic review and meta-analysis of animal studies
Source: EXCLI J. 2024 Jan 3;23:18–33. doi: 10.17179/excli2023-6740 (PMC10864704; doi:10.17179/excli2023-6740)
Supplement: Supplementary information [file EXCLI-23-18-s-001.pdf]

*EXCLI Journal 2024;23:18-33 – ISSN 1611-2156*
*Received: October 29, 2023, accepted: December 18, 2023, published: January 03, 2024*

## Supplementary information to:

### Review article:

# MYOCARDIAL INFARCT SIZE IS REDUCED BY NITRITE AND NITRATE ADMINISTRATION: A SYSTEMATIC REVIEW AND META-ANALYSIS OF ANIMAL STUDIES

Younes Yassaghi[1], Sajad Jeddi[1], Khosrow Kashfi[2], Asghar Ghasemi[1*]

[1] Endocrine Physiology Research Center, Research Institute for Endocrine Sciences, Shahid Beheshti University of Medical Sciences, Tehran, Iran
[2] Department of Molecular, Cellular, and Biomedical Sciences, Sophie Davis School of Biomedical Education, City University of New York School of Medicine, NY, USA

* **Corresponding author:** Dr. Asghar Ghasemi, Endocrine Physiology Research Center, Research Institute for Endocrine Sciences, Shahid Beheshti University of Medical Sciences. No. 24, Parvaneh Street, Yaman Street, Velenjak, Tehran, I.R. Iran. P.O. Box: 19395-4763, Tel: +9821-22432500, Fax: +9821-22416264, E-mail: Ghasemi@sbmu.ac.ir

https://dx.doi.org/10.17179/excli2023-6740

This is an Open Access article distributed under the terms of the Creative Commons Attribution License (http://creativecommons.org/licenses/by/4.0/).

EXCLI Journal 2024;23:18-33 – ISSN 1611-2156
Received: October 29, 2023, accepted: December 18, 2023, published: January 03, 2024

**Supplementary Table 1:** Search strategy

| Database | Step | Search terms |
|---|---|---|
| PubMed | #1 | (("Nitrates"[Mesh]) OR ("Nitrites"[Mesh]) OR ("Nitrogen dioxide"[Mesh]) OR ("Nitric oxide"[Mesh]) OR (“Sodium nitrite” [Title/Abstract]) OR (“Sodium nitrate” [Title/Abstract]) OR (“Beetroot” [Title/Abstract])) |
| | #2 | (("Reperfusion injury"[Mesh]) OR (“Ischemia/reperfusion” [Title/Abstract]) OR (“Ischemia-reperfusion” [Title/Abstract]) OR (“Ischemia reperfusion” [Title/Abstract]) OR ("Myocardial ischemia"[Mesh]) OR (“Myocardial ischemia” [Title/Abstract]) OR (“Cardiac ischemia” [Title/Abstract]) OR (“Heart ischemia” [Title/Abstract]) OR ("Myocardial infarction"[Mesh]) OR (“Myocardial infarction” [Title/Abstract]) OR (“Cardioprotection” [Title/Abstract])) |
| | #3 | #1 AND #2 |
| Scopus | #1 | ((TITLE-ABS-KEY (Nitrate) OR TITLE-ABS-KEY (Nitrite) OR TITLE-ABS-KEY ("Nitrogen dioxide") OR TITLE-ABS-KEY ("Nitric oxide") OR TITLE-ABS-KEY ("Sodium nitrite") OR TITLE-ABS-KEY ("Sodium nitrate") OR TITLE-ABS-KEY (Beetroot)) |
| | #2 | ((TITLE-ABS-KEY ("Reperfusion injury") OR TITLE-ABS-KEY (Ischemia/reperfusion) OR TITLE-ABS-KEY (Ischemia-reperfusion) OR TITLE-ABS-KEY ("Ischemia reperfusion") OR TITLE-ABS-KEY ("Myocardial ischemia") OR TITLE-ABS-KEY ("Cardiac ischemia") OR TITLE-ABS-KEY ("Heart ischemia") OR TITLE-ABS-KEY ("Myocardial infarction") OR TITLE-ABS-KEY (Cardioprotection)) |
| | #3 | #1 AND #2 |
| Web of Science | #1 | (TS=("Nitrate" OR "Nitrite" OR "Nitrogen dioxide" OR "Nitric oxide" OR “Sodium nitrite” OR “Sodium nitrate” OR “Beetroot”)) |
| | #2 | (TS=("Reperfusion injury" OR “Ischemia/reperfusion” OR “Ischemia-reperfusion” OR “Ischemia reperfusion” OR “Myocardial ischemia” OR “Cardiac ischemia” OR “Heart ischemia” OR “Myocardial infarction” OR “Cardioprotection”)) |
| | #3 | #1 AND #2 |

ABS=abstract, KEY=keyword, TS=Topic (including title, abstract, author, and keywords)
